# Opioid prescribing practices following elective surgery in Otolaryngology-Head & Neck Surgery

**DOI:** 10.1186/s40463-019-0352-9

**Published:** 2019-06-24

**Authors:** Mathew Biskup, Agnieszka Dzioba, Leigh J. Sowerby, Eric Monteiro, Julie Strychowsky

**Affiliations:** 1 Division of Otolaryngology-Head & Neck Surgery, Dalhousie University, QEII Health Sciences Centre, 3rd floor Dickson Building, 5820 University Avenue, Halifax, NS B3H 1Y9 Canada; 2 Department of Otolaryngology-Head and Neck Surgery, Schulich School of Medicine and Dentistry, Western University, London, Ontario Canada; 3 Department of Otolaryngology-Head and Neck Surgery, University of Toronto, Toronto, Ontario Canada

**Keywords:** Otolaryngology, Opioid, Narcotic, Post-operative pain, Prescribing patterns, Opioid epidemic, Analgesia

## Abstract

**Background:**

Prescription opioid abuse has become a major issue across the world and especially in North America. Canada has the second highest number of opioid prescriptions per capita in the world, second only to the United States, with numbers continuing to rise in recent years. Surgeons play a critical role in this discussion as they are responsible for the management of post-operative pain in their patients**.** The objective of this study is to evaluate the opioid prescribing practices of Otolaryngologists-Head and Neck Surgeons in Canada and determine factors that may influence these practices.

**Methods:**

The online survey was distributed to members of the Canadian Society of Otolaryngology-Head and Neck Surgery. Questions surveyed the respondents’ demographics and opioid prescribing practices for common pediatric and adult elective surgeries.

**Results:**

The survey was sent to 670 surgeons and trainees and 121 responses were received (18%). There was representation across all subspecialties with a mix of community and academic surgeons. The most commonly prescribed opioid was Codeine/Acetaminophen, 48.2% (*n* = 53), in the adult population, and Morphine, 47.1% (*n* = 41), in the pediatric population. The median total oral morphine equivalents prescribed across all adult surgeries was 123.75 mg (24.75 doses). The surgery with the highest oral morphine equivalents prescribed was tonsillectomy/adenoidectomy for both adult and pediatric patients, with a median of 150 mg (30 doses) for adults and 4.5 mg/kg (23 doses) for pediatrics. Gender, training years, year in residency, or reported level of conservatism did not predict the dose prescribed to either adult or pediatric patients. Due to the relatively low response rate, the generalizability of these results is unclear.

**Conclusions:**

Our study demonstrates a wide variability in opioid prescriptions across procedures and within each individual procedure. This variability reflects the lack of guidelines available for post-operative opioid prescribing and suggests that some Otolaryngologists may be prescribing higher doses of opioids than required. Opportunities for improving patient safety and resource stewardship regarding optimal prescribing practices should be explored.

**Electronic supplementary material:**

The online version of this article (10.1186/s40463-019-0352-9) contains supplementary material, which is available to authorized users.

## Background

Prescription opioid abuse has become an epidemic across the world and especially in North America. Worldwide, Canada is second only to the United States in number of opioid prescriptions per capita, with numbers continuing to increase [1]. This can be attributed, at least in part, to a report released by the Joint Commission on Accreditation of Healthcare Organizations in 2001, which introduced pain as the “fifth vital sign” [2]. Many interpreted this as a recommendation to eliminate pain entirely, leading to a significant increase in opioid use [3]. As the risk of opioid addiction and diversion has become apparent over time, the total amount of opioid prescriptions in the United States has decreased annually since 2012, however, unlike the United States, Canadian prescriptions have continued to rise [4, 5].

In Canada, an estimated 13% of the general population used prescription opioids in 2015, with approximately 2.2% acknowledging opioid abuse. This resulted in an average of 13 opioid overdoses per day requiring hospitalizations and 2816 opioid-related deaths in 2016 [6, 7]. These numbers continue to increase, demonstrating the importance of health care practitioners only prescribing these medications when they are needed.

Surgeons are responsible for approximately 10% of total opioid prescriptions, second only to chronic pain physicians in their proportion of opioid prescriptions [8]. Management of post-operative pain is an important part of peri-operative care, often treated with a short-term course of opioids to ease the recovery of the patient. To date, only general guidelines have been established for the management of post-operative pain control, with surgeons often arbitrarily prescribing the amount of pills required for individual procedures [9]. The surgeon must balance the need to adequately control the occasional experience of severe pain following surgery, while avoiding over-prescription, which is both a cost to the health care system and a danger to public health.

## Methods

This study used an explorative survey designed to investigate post-operative opioid prescribing practices among Otolaryngologists following elective surgeries in Canada. To collect the data, a 22-item questionnaire (Additional file 1) was developed and hosted on the web-based survey platform Qualtrics (version December, 2017. Copyright© 2017 Qualtrics). Development of the survey by the study team was based on critical review of the literature and clinical expertise. The survey was distributed to members of the Canadian Society of Otolaryngology-Head and Neck Surgery (CSO) via email. Eligible participants included members of the CSO actively practicing in Otolaryngology across Canada including residents, fellows, and staff. 670 individuals met the criteria and were distributed a link to the survey, along with the study letter of information and consent. A single reminder email was sent 3 weeks after the initial study invitation to encourage further response.

The survey was designed to take approximately 5 min to complete. Survey items included respondents’ demographics, types of opioids prescribed, typical dosage and number of doses prescribed for common pediatric and adult elective surgeries in Otolaryngology-Head & Neck Surgery. The list of common surgical procedures performed across various subspecialties was formulated and agreed upon by our team. A French translated version of the survey was available to respondents. Survey responses were anonymous. Institutional ethics review board approval for this study was obtained from Western University in London, Ontario, Canada (REB# 111468).

### Data analysis

A descriptive analysis of study outcomes was performed. Frequency data for demographics, types of opioids prescribed, and items with Likert response scales were calculated. All opioid doses were converted to their oral morphine equivalent (OME) doses for further comparison. This conversion was done by multiplying the number of tablets prescribed by the strength of the tablets to get the total dose, then using an opioid conversion chart to find the potency ratio, which is multiplied by the total dose to find the total OME [10]. A separate conversion chart was used for the potency ratio of tramadol as it was not reported in the Canadian guideline [11]. For medications whose doses were reported in mg/kg, an average adult weight of 60 kg was used to calculate total dose in mg for comparison of data. The conversion numbers used along with an example calculation can be found in the Appendix. Pediatric dosing was reported as OME doses in mg/kg and found in a similar manner, which could be found in practice by multiplying the volume of opioid prescribed in mL, by the potency of the medication in mg/mL to obtain the total mg, then dividing the total OME found by the weight of the child to determine OME in mg/kg. Medians and ranges were calculated for opioid doses and number of doses prescribed. In addition to descriptive statistics, several statistical analyses were undertaken. Pearson chi-square tests were conducted to compare differences between residents and consultants and differences between males and females, in rates of opioids prescription for pediatric and adult elective surgeries, revealing no statistically significant differences (*p* > .05). As a result, groups were combined for all main analyses.

Pearson chi-square analyses were conducted to evaluate differences between procedures in rates of opioid prescription. Furthermore, chi-square analyses were also undertaken to compare differences in rates of opioid prescriptions between adult and pediatric surgeries (tonsillectomy and/or adenoidectomy (T&A), tympanoplasty and/or ossiculoplasty and/or mastoidectomy (T/O/M), and, neck excisions).

A comparison of differences in number of doses of opioids prescribed between adult and pediatric procedures could not be performed because doses were reported in terms of average OME prescribed for adult procedures and average OME/kg prescribed for pediatric procedures. However, separate analyses were undertaken to evaluate differences in dosages prescribed between procedures; due to the nonparametric distribution of the data, Wilcoxon signed-rank tests were performed. Finally, linear regression analyses were conducted to determine if gender, training years, year in residency, or level of conservatism were related to number of doses prescribed for adult procedures (i.e., OME) and pediatric procedures (i.e., OME/kg). Data were analyzed using the statistical package for the social sciences (IBM Corp. Released 2017. IBM SPSS Statistics for Windows, Version 25.0. Armonk, NY: IBM Corp). Statistical significance was determined a priori at the alpha level of .05.

## Results

Of the 670 eligible individuals who were emailed the survey, we received 121 responses for a total capture rate of 18%. This included responses from 98 out of 500 (19.6%) consultants and 23 out of 170 (13.5%) residents. Of the 121 responses received, 4 (3.3%) were completed in French. There was a distribution across all Otolaryngology subspecialties and a mix of community and academic surgeons. Full demographic data is reported in Table 1.

**Table 1:** Respondent demographics

Total Respondents	121
Sex	
Male	86 (71.1%)
Female	33 (27.3%)
Undisclosed	2 (1.7%)
Level of Training	
Consultants	98 (81.0%)
Residents	23 (19.0%)
PGY1	2 (8.7%)
PGY2	2 (8.7%)
PGY3	4 (17.4%)
PGY4	5 (21.7%)
PGY5	8 (34.8%)
Practice Setting	
Academic	51 (43.6%)
Community	33 (28.2%)
Both	33 (28.2%)
Practice Area	
General Otolaryngology	90 (74.4%)
Head and Neck Surgery	51 (42.1%)
Pediatric Otolaryngology	45 (37.2%)
Rhinology	45 (37.2%)
Otology/Neurotology	43 (35.5%)
Facial Plastics and Reconstructive Surgery	30 (24.8%)
Laryngology	28 (23.1%)
Multiple Areas	77 (63.8%)
Patient Population	
Pediatric	7 (6.0%)
Adult	30 (25.6%)
Both	80 (68.4%)

**Fig. 1:**
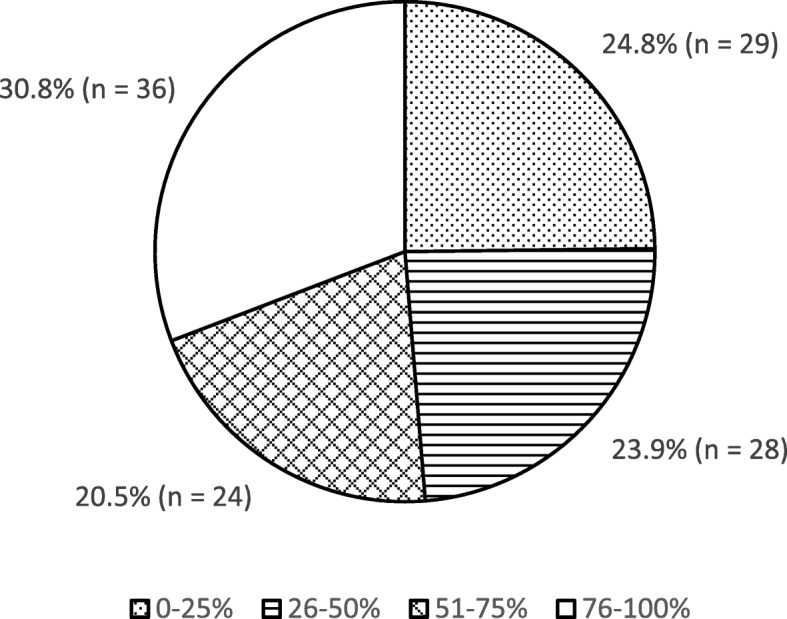
Proportion of respondents who prescribed opioids to each percentage range of patients post-operatively

**Fig. 2:**
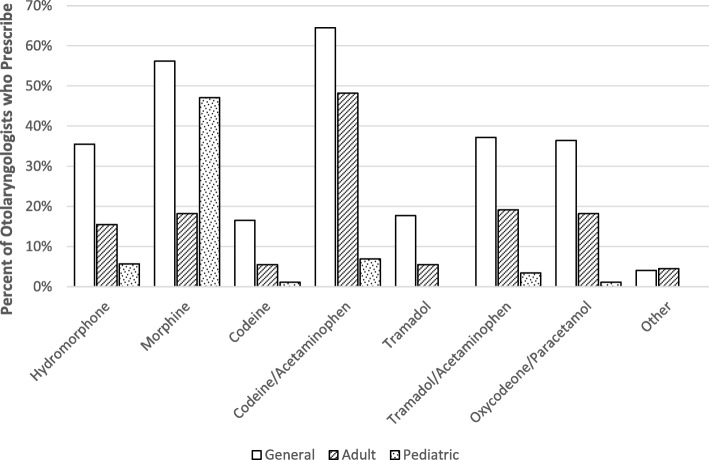
Distribution of specific opioid prescriptions by respondents

There was a wide distribution in the percentage of patients prescribed opioids by the respondents as displayed in Fig. 1. 84.5% (*n* = 98) of respondents endorsed prescribing a variety of opioids, while the remaining 15.5% (*n* = 18) routinely prescribe only one type of opioid. 76.8% (*n* = 73) respondents reported mechanisms in place at their medical center to track whether prescriptions had been filled. When asked if respondents believed that opioids are overused, 8.2% (*n* = 8) strongly disagreed, 23.7% (*n* = 23) somewhat disagreed, 20.6% (*n* = 20) neither agreed nor disagreed, 34.0% (*n* = 33) somewhat agreed and 13.4% (*n* = 13) strongly agreed. Regarding respondents’ self-reported degree of conservatism in opioid prescribing, with 1 being ‘not at all conservative’ and 10 being ‘extremely conservative’, responses ranged from 2 to 10 with a mean (SD) of 5.98 (2.18). Specific opioids were used by respondents in various frequencies, with the most widely used opioid being Codeine/Acetaminophen with 64.5% (*n* = 78) of respondents, followed by Morphine with 56.2% (*n* = 68). For respondents who serve the adult population (*n* = 110), the most commonly prescribed opioid was Codeine/Acetaminophen at 48.2% (*n* = 53), while Morphine was the most common opioid prescribed by respondents who serve the pediatric population at 47.1% (*n* = 41/87). The full distribution of opioids used can be seen in Fig. 2.

Results of opioid prescribing were analyzed based on adult versus pediatric populations. Of respondents who served both pediatric and adult populations, respondents were statistically significantly more likely to prescribe opioids to adult than pediatric patients for T&A procedures (*χ*^2^ = 5.74, *p* = .02), and T/O/M procedures (*χ*^2^ = 4.87, *p* = .03) but not for neck excision procedures (*χ*^**2**^ = 2.96, *p* = .09). Of the surgeries performed in an adult population, the median total OME prescribed was 124 mg corresponding to 25 × 5 mg tablets of Morphine. Data for the proportion of respondents that did not prescribe opioids for each adult procedure is reported in Table 2; statistically significantly difference between all pairs of adult procedures were found. (*χ*^**2**^ = 7.53 to 73.67; *p* = <.01 to <.001).

**Table 2:** Adult surgery opioid use

Procedure	Percent of respondents not using Opioids (N/^a^)
All Adult Procedures	2.7 (3/110)
Tonsillectomy and/or Adenoidectomy	1.2 (1/81)
Tympanoplasty and/or Ossiculoplasty and/or Mastoidectomy	20.6 (13/63)
Septoplasty	14.3 (12/84)
Septorhinoplasty	12.1 (7/58)
Functional Endoscopic Sinus Surgery	12.3 (10/81)
Thyroidectomy	8.1 (5/62)
Parotidectomy	11.9 (8/67)
Excision of Skin Lesion +/− Flap Reconstruction	39.4 (28/71)

^a^Number of respondents who serve the adult population and perform this procedure

**Fig. 3:**
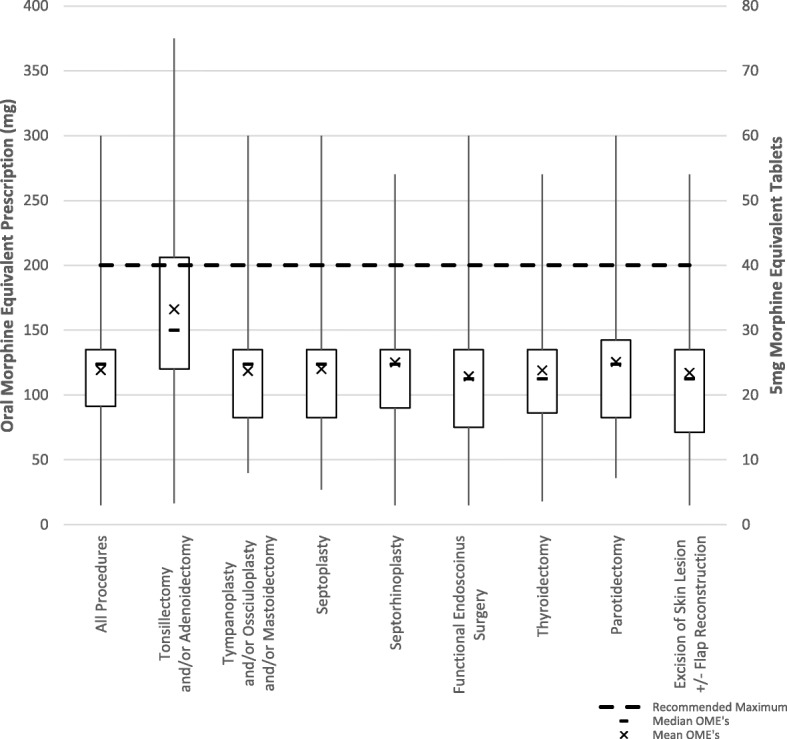
Discharge opioid prescribing across various adult Otolaryngology elective procedures

The surgery with the highest total OME was a tonsillectomy and/or adenoidectomy, with a median of 150 mg (30 tablets or doses) OME and an interquartile range from 120 mg (24 tablets) to 206.25 mg (41 tablets), while the lowest prescriptions were seen in functional endoscopic sinus surgeries with a median of 112.5 mg (23 tablets) OME and an interquartile range from 75 mg (15 tablets) to 135 mg (27 tablets). Differences in median OME opioid prescribed for adult tonsillectomy and/or adenoidectomy were statistically significantly higher than all other adult elective surgeries surveyed (Z = − 3.81 to − 4.64, *p* < .001). In addition, median OME opioid prescribed for septorhinoplasty procedures were statistically significantly higher than FESS procedures (Z = − 2.52, *p* = .01). All other pairwise comparisons of OME opioid prescriptions between adult procedures were statistically nonsignificant (*p* > .05). The distribution of responses for each adult surgery can be seen in Table 3 and Fig. 3.

**Table 3:** Adult surgery opioid prescribing

Procedure	N = # of responses	Median Total OME [mg] (^a^)	Total OME Inter-quartile Range (Q1-Q3) [mg] (^a^)	Total OME Range [mg] (^a^)
All Adult Procedures	81	123.75 (25)	43.75 (9)	15–300 (3–60)
Tonsillectomy and/or Adenoidectomy	63	150 (30)	86.25 (17)	16.5–375 (3–75)
Tympanoplasty and/or Ossiculoplasty and/or Mastoidectomy	36	123.75 (25)	52.50 (11)	40–300 (8–60)
Septoplasty	54	123.75 (25)	52.50 (11)	27–300 (5.4–60)
Septorhinoplasty	38	123.75 (25)	45 (9)	15–270 (3–54)
Functional Endoscopic Sinus Surgery	53	112.5 (23)	60 (12)	15–300 (3–60)
Thyroidectomy	47	112.5 (23)	48.75 (10)	18–270 (3.6–54)
Parotidectomy	50	123.75 (25)	59.90 (12)	36–300 (7.2–60)
Excision of Skin Lesion +/− Flap Reconstruction	30	112.5 (23)	63.75 (13)	15–270 (3–54)

^a^5 mg Morphine Equivalent Tablets

For pediatric surgeries, 83% (*n* = 73) of respondents reported using medications with per kilogram dosing, including Morphine and Hydromorphone, while the remainder used medications with set tablet doses including Codeine, Codeine/Acetaminophen, Tramadol/Acetaminophen and Oxycodone/Paracetamol. For each individual surgery, a set of respondents reported not prescribing opioids as seen in Table 4; differences in rates of no opioid use were statistically significant between all pairs of pediatric procedures (*χ*^**2**^ = 28.90 to 46.70; p < .001).

**Table 4:** Pediatric surgery opioid use

Procedure	Percent of respondents not using Opioids (N/^a^)
All Pediatric Procedures	17.2 (15/87)
Tonsillectomy and/or Adenoidectomy	42.2 (35/83)
Tympanoplasty and/or Ossiculoplasty and/or Mastoidectomy	62.7 (32/51)
Excision of Neck Mass	46.6 (27/58)

^a^Number of respondents who serve the pediatric population and perform this procedure

**Fig. 4:**
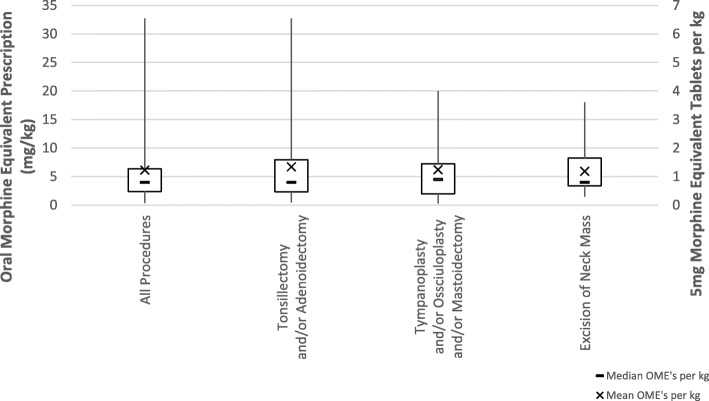
Discharge opioid prescribing across various pediatric Otolaryngology elective procedures

All data below is reported for medications using per kilogram dosing due to the low number of responses for tablet-dosing. Across all surgeries, the median OME was 4 mg/kg with an interquartile range of 2.38 to 6.38 mg/kg. The highest median OME/kg was reported for T&A at 4.5 mg/kg with an interquartile range of 2.31 to 7.97 mg/kg. T/O/M and, excision of a neck mass shared a median of OME/kg at 4 mg/kg and a range of 2.00 to 7.25 mg/kg and, 3.38 to 8.25 mg/kg respectively. Differences in median OME/kg opioid prescriptions between T&A and both T/O/M and excision of a neck mass were statistically significant (Z = − 2.03 to − 2.61, *p* = .01 to .04). The results across all surgeries as well as those for tablet-based prescriptions can be found in Tables 5 and 6 and Fig. 4.

**Table 5:** Pediatric surgery opioid prescribing (weight-based medications)

Procedure	N = # of responses	Median OME [mg/kg]	Total OME Inter-quartile Range (Q1-Q3) [mg/kg]	OME Range [mg/kg]
All Pediatric Procedures	45	4	4.00	0.38–32.75
Tonsillectomy and/or Adenoidectomy	40	4.5	5.66	0.45–32.75
Tympanoplasty and/or Ossiculoplasty and/or Mastoidectomy	12	4	5.25	0.3–20
Excision of Neck Mass	21	4	4.87	1.5–18

**Table 6:** Pediatric surgery opioid prescribing (non weight based medications)

Procedure	N = # of responses	Median OME [mg] (^a^)	Total OME Inter-quartile Range (Q1-Q3) [mg] (^a^)	OME Range [mg] (^a^)
All Pediatric Procedures	10	82.5 (16.5)	59.69 (12)	13.8–180 (3–36)
Tonsillectomy and/or Adenoidectomy	6	123.75 (24.75)	103.44 (21)	13.8–180 (3–36)
Tympanoplasty and/or Ossiculoplasty and/or Mastoidectomy	4	78.75 (15.75)	121.88 (24)	20–180 (4–36)
Excision of Neck Mass	5	90 (18)	73.12 (15)	78.8–180 (16–36)

^a^5 mg Morphine Equivalent Tablets

Regression analyses were undertaken to look for factors that may influence the amount of opioids prescribed for adult and pediatric procedures. Simple linear regression models indicated that gender, training years, year in residency, and reported level of conservatism did not predict the OME dose prescribed to either adult or pediatric patients (*p* > .05 for all regression models).

## Discussion

Surgeons may often be tempted to prescribe higher quantities of opioids than required to avoid the possibility of patients returning due to inadequate pain management. This practice can place patients in danger of developing a dependence to these medications which can result in chronic use [12, 13]. A recent review article reported that anywhere from 67 to 92% of surgical patients reported unused opioids following surgery, with very low rates of safe storage or disposal [14]. Similarly, a study published in 2017 by Thiels et al., looked at opioid prescriptions among 7651 patients undergoing a variety of elective surgeries and found that over 80% of patients were overprescribed opioids [15]. These excess pills can occasionally be diverted into the illicit market or taken by adolescents and young adults in the home for recreational use [16, 17].

Previous studies have been carried out to document prescribing practices of surgeons post-operatively in the fields of Orthopedics, General Surgery, Obstetrics, Oral Maxillofacial Surgery and Dermatology [18–22]. Most recently this has also become a hot topic in the field of Otolaryngology - Head and Neck Surgery with multiple papers published on the topic within the last year [23–27]. These studies looked at opioid prescribing practices in the United States, however there have been no studies published examining opioid use in Canada. The present paper aims to fill this gap in literature, to evaluate the prescribing habits of Otolaryngologists in Canada, and determine factors that may influence prescribing practices.

Due to the relatively low response, the generalizability of these results is unclear; however, our results suggest that there is significant variability in opioid prescribing practices across all elective procedures for both adult and pediatric patients in Otolaryngology - Head and Neck Surgery in Canada. There is an ongoing epidemic of opioid use with associated dangers such as addiction and overdose, yet there is a lack of established procedure-specific and age-specific standards or guidelines for physicians to follow. This is evidenced by the similar opioid prescribing amounts across a range of dissimilar procedures with expectantly differing amounts of pain, as seen in our results. Physicians and surgeons often estimate appropriate and safe dosing for their patients and may not receive feedback from their patients regarding how many pills were actually consumed and whether their pain management was adequate. This variability is not unique to Otolaryngology - Head and Neck Surgery. Recent studies in the areas of upper extremity surgery, Maxillofacial surgery, hand surgery, Urology and General surgery consistently show wide variability across different procedures and within each individual procedure [19, 28–31]. Often out of convenience to both the patient and the physician, the amount of opioids prescribed may be overestimated to ensure that the patient will not need to refill their prescription or present to the emergency department for uncontrolled pain. This thinking may be flawed however, as the inherent risks of overprescribing seem to outweigh the benefits of overprescribing. Studies have suggested that up to 70% of opioid pills prescribed post-operatively go unused [19, 28]. Hill and colleagues (2017) estimated that the amount of opioids prescribed could be decreased by 43% if they allowed for the possibility of up to 20% of their general surgery patients to require refill. Operationalizing this idea, another study by Hill et al. (2018) used an educational intervention for physicians, encouraging the use of non-steroidal anti-inflammatories and acetaminophen, and decreased opioid prescription amounts. This resulted in a 50% decrease in prescriptions with only a 0.4% refill rate during their study [32]. Within Otolaryngology - Head and Neck Surgery, a recent US national database study examining ambulatory opioid prescribing found that approximately 1 in 133 patients are seen in ambulatory clinic regarding post operative pain management that requires an opioid prescription [23].

A similar survey study design by Schwartz et al. (2018) was recently published that examined opioid prescribing of Otolaryngologists in the United States [24]. The procedure with the highest opioid prescriptions was similarly tonsillectomy, with a mean of 37 tablets (similar to the reported mean of 33 tablets in this paper). The remainder of the procedures had similar results to ours, however the American study had more variability between procedures including tympanoplasty, mastoidectomy and soft tissue excision, which had lower rates of prescription opioids compared to our study. In regards to specific opioids used, the American study found that the most commonly prescribed opioids were Hydrocodone followed by Oxycodone, in contrast with Codeine/Acetaminophen and Morphine being the most commonly prescribed in our study. The American result is consistent with other studies examining post operative opioid prescribing in the United States [19, 25]. In Canada however, Hydrocodone is only approved for treatment of cough as opposed to pain control and Health Canada has warned against its use in children and adolescents [33, 34]. This, along with different national prescribing trends, likely explains the discrepancy in our results.

Concerningly, this study found that codeine was still being used in children despite FDA and Health Canada warnings about its use in children. Codeine is metabolized in the cytochrome P450 pathway in the liver and it has been found that codeine can be metabolized differently by different individuals, with some being ultra-rapid metabolizers and others being poor metabolizers [35]. This creates an inconsistent medication effect profile and can be dangerous in children leading to possible overdoses in those known as ultra-rapid metabolizers [36]. A recent study by Chua et al. (2017) looked specifically at the use of Codeine in pediatric T&A and found that although there had been a drop in codeine prescriptions following an FDA warning in 2012, approximately 5.1% of children received codeine for pain control following T&A in 2015 [26]. Health Canada recommends against the use of Codeine in children under 12 [34]. The FDA previously had a similar recommendation against the use of codeine in patients under age 12, however the recommendation was amended to include patients up to age 18 as part of a safety announcement released in January 2018 [37]. In our study, we found that 8% (*n* = 7) of the respondents continue to prescribe codeine to pediatric patients (Codeine 1.1% (*n* = 1) and Codeine/Acetaminophen (*n* = 6)). Of these responses, all but one for Codeine/Acetaminophen indicated its use only in the adolescent population. Unfortunately, the threshold for the older versus younger children was not specified in the survey, so it is unclear whether the remaining 6 respondents were prescribing to children below the age of 12. Another concern, discussed in the CDC paper by Shah et al. (2017) was the use of tramadol and it's risk of long term dependency [38]. The study found that there was a 13.7% risk of chronic use at 1 year with tramadol compared to short-acting opioids other than hydrocodone and oxycodone at 8.9% and hydrocodone and oxycodone the lowest at around 5%. This result may be surprising as tramadol was thought to be relatively safer due to its lower affinity for μ-receptors [39]. This thinking may explain the relatively high use of tramadol we saw in our study, either alone or in combination with acetaminophen. However, this prescribing practice may bring about an unintended risk onto our patients. Despite the CDC warning against the use of tramadol in children under 12 as well as the FDA and Health Canada warnings suggesting tramadol should not be used in patients under 18, there remained respondents that used this medication in the pediatric population [36, 40, 41]. Lastly, it is important to note that opioids, at the appropriate doses, are both safe and effective after pediatric surgeries such as T&A’s and remain an important part of post-operative care [42].

To date, there has been very little research or guidance on the requirement for opioids in elective Otolaryngologic procedures. One study by Patel et al. (2018) looked specifically at opioid prescribing post-rhinoplasty and found that of the typical 20 to 30 hydrocodone tablets initially prescribed, a mean of only 8.7 were consumed, with approximately 75% of the patients consuming less than 15 [27]. Although just one procedure, this demonstrates the discrepancy in the amount of opioids prescribed compared to what is actually used by the patients.

In a clinical practice guideline from Minnesota discussing opioid use for acute pain, they recommended that no prescriptions should exceed 200 OME or 7 days [43]. This corresponds with a CDC guideline in 2016 recommending no more than 7 days with sharply increased risks of long-term use after 5 days [44]. Similarly, a quality standard by Health Quality Ontario suggests that 3 days of opioids is often sufficient and greater than 7 days is rarely indicated [45]. In our study, the interquartile range for all procedures fell below this limit with the exception of T&A, which crossed just above this recommended limit. This information corresponds to another study by Thiels et al. (2017), which looked at the variations in opioid prescribing in various procedures across multiple specialties and found that the only procedures that fell below the 200 OME limit were those involving the neck [15]. Whether this speaks to opioid prescribing practices of Otolaryngologists, minimal pain following Otolaryngology procedures or a mix of both is unclear, however this demonstrates that current prescribing practices may not be far off what is recommended.

Our results are limited by our response rate (18%) and our study design, which asked respondents to estimate their usual prescriptions for each procedure. This does not take into account individual patient factors, which may influence prescribing practices. We did not obtain information on other pain management strategies used including non-opioid analgesics and were not able to determine the percentage of opioids used by patients. It should also be noted that resident prescribing practices may be highly influenced by practices of the consultants under which they work rather than individual preference. This could explain why resident prescribing closely matched that of consultants and may have resulted in the practices of consultants who work in academic centers with residents to be over represented compared to what is reported.

## Conclusions

This study shows a wide variability in both the types of opioids prescribed as well as the amount of opioids prescribed for adult and paediatric elective procedures in Otolaryngology - Head and Neck Surgery. The majority of opioids prescribed seem to be within recommended maximums, but these maximums are generic and not procedure-specific. Some Otolaryngologists are continuing to prescribe certain opioids despite emerging evidence of harm regarding medications, such as codeine and oxycodone in the pediatric population and possibly tramadol in the adult population. Further research and quality improvement interventions should be focussed on guiding Otolaryngologists on appropriate prescribing practices with goals of improving patient safety, reducing opioid over-prescription, and guiding appropriate resource stewardship.

### Additional file

Additional file 1: Opioid prescribing survey (DOCX 18 kb)

## Data Availability

The datasets used and/or analyzed in this study are available from the corresponding author on reasonable request.

## Appendix

**Table 7:** Opioid conversions

Oral Morphine Equivalent Conversions [10, 11]		
Opioid	Potency Ratio (Opioid: OME)	OME to Opioid Ratio
Morphine	1	1
Codeine	0.15	6.67
Tramadol	0.15	6.67
Oxycodone	1.5	0.67
Hydromorphone	5	0.2

Conversion Example

If a prescription was Hydromorphone 1 mg PO q4h prn, Dispense: 30 (thirty) × 1 mg tablets, it could be converted to total OME as follows: Total Dose = # of tablets x strength of tablets = 30 × 1 = 30 mg Hydromorphone. In the conversion table, the potency ratio for hydromorphone is 5, thus Total OME = Total Dose x potency ratio = 30 × 5 = 150 mg OME.

## Abbreviations

CSO: Canadian Society of Otolaryngology-Head and Neck Surgery
OME: Oral Morphine Equivalent
T&A: Tonsillectomy and/or Adenoidectomy
T/O/M: Tympanosplasty and/or Ossiculoplasty and/or Mastoidectomy
