# Supplementary material for: Opioid prescribing practices following elective surgery in Otolaryngology-Head & Neck Surgery
Source: J Otolaryngol Head Neck Surg. 2019 Jun 24;48:29. doi: 10.1186/s40463-019-0352-9 (PMC6591868; doi:10.1186/s40463-019-0352-9)
Supplement: Supplementary file 1 — Opioid prescribing survey (DOCX 18 kb) [file 40463_2019_352_MOESM1_ESM.docx]

Appendix A – Opioid Prescribing Survey

- 1. Opioid Prescription Practices Following Elective Surgery in Otolaryngology-Head & Neck Surgery
  2. The following is a survey of opioid prescribing practices in Otolaryngology-Head & Neck Surgery across Canada. The survey consists of 22 items.  This survey will take approximately 5 minutes to complete. Please click on the "Next" button below to start the survey.
  3. Q1 What is your gender?
  4. Male
  5. Female
  6. Prefer not to disclose
  7. Q2 What Otolaryngology position do you currently hold?
  8. Consultant
  9. Fellow
  10. Resident
  11. Q3 How many years have you been in practice, not including your training years (please enter 0 if less than a year): _____________________
  12. Q4 What year in residency are you currently in?
  13. Year 1
  14. Year 2
  15. Year 3
  16. Year 4
  17. Year 5
  18. Q5 What type of setting do you currently practice in?
  19. Academic
  20. Community
  21. Both
  22. Q6   What are your current areas of practice? (check all that apply)
  23. General Otolaryngology
  24. Facial Plastic and Reconstructive Surgery
  25. Head and Neck Surgery
  26. Laryngology
  27. Otology/Neurotology
  28. Pediatric Otolaryngology
  29. Rhinology
  30. Q7 What is the average number of elective surgeries you perform per month?_____________
  31. Q8 What proportion of patients that undergo elective surgery do you prescribe an opioid to:

0 to 25%

26 to 50%

51 to 75%

76 to 100%

Q9 What patient age group(s) do you serve in your practice?

Pediatric

Adult

Both

Q10 Which of the following opioids do you prescribe to patients following elective surgery (Please check all that apply):

Codeine

Dilaudid

Morphine

Percocet

Tramacet

Tramadol

Tylenol#3

Other

Q11 Please enter up to three other opioids you prescribe following elective surgery: _________

Q12 Which of the following **pediatric** surgeries do you perform in your practice (check all that apply):

Tonsillectomy and/or adenoidectomy

Tympanoplasty and/or ossiculoplasty and/or mastoidectomy

Excision of congenital neck masses

Q13 Does the dosage (i.e., mg/kg/day) and/or type of opioid you prescribe to pediatric patients following elective surgery **depend** on the patient's age?

Yes

No

Q14 What is the age cut-off that you use to determine opioid dosage?  Please enter the cut-off age. (For example, if you prescribe lower doses to children who are younger than 5 years of age, and higher doses to children who are 5 years and older, you would enter 5 below)

Cut-off age: __________

Q15 For each of the pediatric elective surgeries listed below, please indicate the **usual**dosage (in mg/kg/day) and **number of doses** for each opioid you prescribe to children for home. Please enter "N/A" if opioid is not prescribed for a given surgery.

Q16 Which of the following **adult** surgeries do you perform in your practice (check all that apply):

Tonsillectomy and/or adenoidectomy

Tympanoplasty and/or ossiculoplasty and/or mastoidectomy

Septoplasty

Septorhinoplasty

Functional endoscopic sinus surgery

Thyroidectomy

Parotidectomy

Excision of skin lesion with/without flap reconstruction

Q17 For each of the adult elective surgeries listed below, please indicate the usual dosage (in mg/kg/day) and number of doses, for each opioid you prescribe to patients for home. Please enter "N/A" if opioid is not prescribed for a given surgery.

Q18 Are there mechanisms in place at your medical center to track whether opioid prescriptions have been filled?

Yes

No

Q19 If yes, please specify the mechanisms in place: ___________________________

Q20 To the best of your knowledge, what percentage of the filled prescription do patients typically use following an elective surgical procedure? For example, if you prescribe 100 doses and the patient uses 75 doses, you would answer 51 to 75%.

0 to 25%

26 to 50%

51 to 75%

76 to 100%

Don't know/Unsure

Q21 In general, do you believe that opioids are overused to manage pain following elective surgical procedures in otolaryngology?

Strongly Disagree

Somewhat disagree

Neither agree nor disagree

Somewhat agree

Strongly agree

Q22 Please indicate how conservative you are with prescribing opioids to manage post-operative pain following elective surgery. Degree of conservatism: 1 -10
